# The National Clinical Assessment Tool for Medical Students in the Emergency Department (NCAT-EM)

**DOI:** 10.5811/westjem.2017.10.34834

**Published:** 2017-12-22

**Authors:** Julianna Jung, Douglas Franzen, Luan Lawson, David Manthey, Matthew Tews, Nicole Dubosh, Jonathan Fisher, Marianne Haughey, Joseph B. House, Arleigh Trainor, David A. Wald, Katherine Hiller

**Affiliations:** * Johns Hopkins University, Department of Emergency Medicine, Baltimore, Maryland; † University of Washington, Department of Emergency Medicine, Seattle, Washington; ‡ East Carolina University, Department of Emergency Medicine, Greenville, North Carolina; § Wake Forest University, Department of Emergency Medicine, Winston-Salem, North Carolina; ¶ Medical College of Georgia, Department of Emergency Medicine, Augusta, Georgia; || Beth Israel Deaconess Medical Center/Harvard Medical School, Boston, Massachusetts; # University of Arizona, Phoenix, Department of Emergency Medicine, Phoenix, Arizona; ** City University of New York, Department of Emergency Medicine, New York, New York; †† University of Michigan, Department of Emergency Medicine, Ann Arbor, Michigan; ‡‡ University of South Dakota, Department of Emergency Medicine, Vermillion, South Dakota; §§ Temple University, Department of Emergency Medicine, Philadelphia, Pennsylvania; ¶¶ University of Arizona, Department of Emergency Medicine, Tucson, Arizona

## Abstract

**Introduction:**

Clinical assessment of medical students in emergency medicine (EM) clerkships is a highly variable process that presents unique challenges and opportunities. Currently, clerkship directors use institution-specific tools with unproven validity and reliability that may or may not address competencies valued most highly in the EM setting. Standardization of assessment practices and development of a common, valid, specialty-specific tool would benefit EM educators and students.

**Methods:**

A two-day national consensus conference was held in March 2016 in the Clerkship Directors in Emergency Medicine (CDEM) track at the Council of Residency Directors in Emergency Medicine (CORD) Academic Assembly in Nashville, TN. The goal of this conference was to standardize assessment practices and to create a national clinical assessment tool for use in EM clerkships across the country. Conference leaders synthesized the literature, articulated major themes and questions pertinent to clinical assessment of students in EM, clarified the issues, and outlined the consensus-building process prior to consensus-building activities.

**Results:**

The first day of the conference was dedicated to developing consensus on these key themes in clinical assessment. The second day of the conference was dedicated to discussing and voting on proposed domains to be included in the national clinical assessment tool. A modified Delphi process was initiated after the conference to reconcile questions and items that did not reach an a priori level of consensus.

**Conclusion:**

The final tool, the National Clinical Assessment Tool for Medical Students in Emergency Medicine (NCAT-EM) is presented here.

## INTRODUCTION

Clinical assessment of medical students in the emergency department (ED) is a highly variable process in which emergency medicine (EM) clerkship directors (CDs) use institution-specific tools that often lack validity evidence,^1^ making it impossible to reliably measure students’ performance or compare students across institutions. Complicating the problem, EM is taught at multiple points in the medical school curriculum (third vs. fourth year); it may be mandatory, elective, or selective; and students seeking careers in EM typically complete clerkships at multiple different institutions.^2^,^3^ Furthermore, some institutions use the same tool for all clerkships, regardless of specialty, an approach that fails to address the unique opportunities, challenges, and priorities inherent to the specialty of EM.^1^

Clinical assessment data is translated into grades, medical student performance evaluations (MSPE, or “dean’s letters”), and the standardized letter of evaluation (SLOE), a critical element of residency application in EM.^4^ The SLOE was developed as a means to discriminate between candidates, and to compare candidates across institutions.^4^,^5^ However, each institution uses its own idiosyncratic approach to collecting, analyzing, and interpreting assessment data, which are derived from highly variable institutional tools that may or may not address the knowledge, skills, and attributes most valued in the EM setting. Grades and SLOEs are key determinants of residency placement, and may dictate whether a student matches into EM at all.^4^ It is thus imperative to ensure the reliability and validity of the assessment process in the interest of students and residency programs alike.

Adoption of a common, specialty-specific assessment tool and standardization of assessment practices across institutions would permit EM CDs to better measure student performance, improve the quality of formative feedback, monitor student progression over time, and compare students across institutions during the residency application process. To this end, a national consensus conference on clinical assessment of medical students in EM clerkships was held in the Clerkship Directors in Emergency Medicine (CDEM) track of the Council of Emergency Medicine Residency Directors (CORD) Academic Assembly in Nashville, TN, in March 2016. The goal of this conference was to develop a standardized clinical assessment tool and guidelines for its use in EM clerkships, based on expert consensus among a national group of EM educators.

## METHODS

### Pre-conference Work

#### Themes of Assessment

Prior to the conference, overarching “themes” surrounding the clinical assessment of medical students were derived from small-group discussions among the executive committee, and refined at a large-group planning meeting in the CDEM track at the 2015 CORD Academic Assembly in Phoenix, Arizona. The themes were not directly related to construction of the final assessment tool – instead, the goal was to clarify the philosophical underpinnings of clinical assessment in the ED, and to identify “best practices” for the acquisition and use of assessment data. The themes identified for consensus discussion were the following:

1. Criterion- vs. normative-referenced assessment
2. Assessment of learners at different levels
3. Translation of end-of-shift assessment data into other products (SLOEs, grades, MSPEs)
4. Implementation and use of assessment tools

The executive committee identified “theme leaders” one year prior to the consensus conference (Table 1). Each theme leader was tasked with recruiting relevant stakeholders to their group, synthesizing the literature on their topic, and articulating key questions pertinent to their theme. Theme leaders were encouraged to participate in other themes’ discussions to assure complete and non-duplicative efforts.

#### Domains of Assessment

In addition to the themes listed above, the executive committee developed a list of potential assessment domains to be considered for inclusion in the assessment tool itself. For each domain, the executive committee drafted a document using a standard format including background information, an operational definition of the domain, a list of possible benefits, drawbacks, and alternatives to including the domain in the final assessment tool, and example items that could potentially be used to assess the domain in question. To the greatest extent possible, these documents were grounded in foundational source materials reflecting national expert consensus regarding each domain.^1^,^4^,^6^–^10^ The purpose of these documents was to highlight key issues within each domain, to standardize items for discussion, and to facilitate rapid construction of an assessment tool based on the sample items within each domain selected for inclusion.

The conference was widely publicized to EM CDs, residency directors, deans, and non-physician educators. When participants registered for the 2016 CORD Academic Assembly, they were invited to register for the consensus conference simultaneously. One week prior to the conference, the executive committee electronically distributed preparatory materials to all registered attendees, including theme summaries, domain descriptions, and reference lists.

### Consensus Conference

#### Day 1

The first day of the conference focused on the overarching themes in clinical assessment. Participants were divided into four small groups. Theme leaders rotated at timed intervals among each of the four groups. Small groups maximized the opportunity for each attendee to actively participate in the discussion. Attendance for each group was recorded, and scribes documented the discussion. After discussion, participants were asked to vote on key questions identified in advance by the theme leaders. Voting was tallied by paper ballot.

#### Day 2

The second day of the conference began with a recap and synthesis of the findings from the first day. Next, the potential domains of assessment were discussed one by one in a large group with all participants, and then participants voted electronically on these questions:

1. Should this domain be included on a national clinical assessment tool?
2. If yes, would the domain best be assessed via a narrative response, a dichotomous response, or a rating scale?
3. Should the example item for the domain be adopted as written, or should it be modified?
4. What modifications, if any, are needed for the item?

The Poll Everywhere electronic audience response system ( www.polleverywhere.com ) was used for voting and to obtain free-text responses for the last question. Additionally, a scribe recorded discussion within the large group. Prior to the conference, the executive committee decided a two-thirds supermajority would constitute consensus.

#### Post-conference Work: The Delphi Process

Following the conference, results were analyzed and reported to the theme leaders and participants. The results were additionally disseminated at the Society of Academic Emergency Medicine Annual Meeting in New Orleans in April 2016. A modified Delphi process was subsequently used to refine and finalize the work of the conference. All conference participants as well as members of CORD and CDEM were invited to participate in the Delphi group, the goals of which were the following:

1. Address unresolved differences regarding themes of assessment
2. Finalize the domains to be included on the national assessment tool
3. Refine the items used to assess each domain
4. Determine design elements for the national assessment tool.

The modified Delphi process spanned several months and included a group of 66 EM educators, including 36 CDs, seven undergraduate medical education directors, 14 assistant/associate program directors, 10 program directors, and four with deanships. The Delphi group used Qualtrics (www.qualtrics.com) to vote on discrete questions and to provide qualitative feedback. Through an iterative process, the group achieved the two-thirds supermajority required for consensus regarding most outstanding questions. Once consensus was achieved on all questions of content, the group conducted a series of web-based teleconferences to address items that did not achieve consensus, and to finalize the wording and design elements of the assessment tool.

## RESULTS

### Participants

A total of 64 people participated on Day 1, including 36 CDs, 25 residency program directors and assistant/associate program directors, eight undergraduate medical education directors, and four with deanships. A total of 76 people participated on Day 2, including 55 CDs, eight residency program directors and assistant/associate program directors, four general teaching faculty, four students, three clerkship coordinators, one resident, and one cognitive psychology expert. Many participants hold more than one role but were asked to list their primary role.

#### Day 1

##### Theme 1: Criterion- vs Norm-referenced Assessment

Half of all participants (51%) favored incorporating elements of both assessment approaches; 37% preferred competency-based assessment only, and 11% preferred norm-based assessment only. This theme also included a discussion of the goals of clinical assessment in the ED. Provision of learner feedback was felt to be the most important goal of assessment by 80% of participants, with generation of grades (36%) and ranking of students for residency application (15%) coming in second and third, respectively.

##### Theme 2: Learners at Different Levels of Training

Participants felt that one assessment tool should be used for all student learners regardless of year of training (67.2%), and that if a clerkship takes multiple levels of students (M3, M4, etc.) all evaluator types (intern, resident, faculty, etc.) should be allowed to assess all levels of learners (91.2%). There was no consensus as to whether grading criteria should differ between third- and fourth-year students (41.4% yes, 58.6% no); but participants agreed that experience level of the student within a given year of training should not affect grading (33.3% yes, 66.7% no).

##### Theme 3: Translation of Clinical Assessment Data into Other Products

Participants agreed that data from a series of rating scale items used across multiple specific domains of assessment could be translated into a final rotation grade (66.7%). However, there was no consensus on whether a single global assessment item could be used independently to generate a final rotation grade (60.0% yes, 23.3% no, 16.7% unsure). There was strong consensus that clinical assessment data should be used to generate grades (83.1%), SLOEs (81.3%) and to determine clinical competency (88.1%); 71.7% agreed that clinical assessment data could and should be incorporated into the SLOE in a standardized manner for all EM-bound students.

##### Theme 4: Issues Around Implementation and Use of Clinical Assessment Tools

Participants agreed that a single assessment tool could be used to measure performance across multiple institutions (82.6%). There was also strong consensus that the unit of observation used for an assessment form should be a single ED shift (84.7%). When asked how many assessments would be necessary to generate a final grade, 94% of respondents indicated that more than five were needed, with two thirds of the responses falling between six and ten shifts.

Participants were unanimous that EM faculty and senior EM residents should be allowed to assess students. However, only a minority felt that assessment should be conducted by junior residents or interns (33.9%), non-physician providers (22%), or off-service residents (3%). The majority (84.7%) agreed assessors should undergo some form of training before assessing students.

#### Day 2

Of the 16 potential domains of assessment presented on Day 2, the group agreed to include nine (agreement 69–98%), and exclude five (agreement 74–90%). Consensus was not reached on two domains (Table 2). Importantly, the group did not feel that the excluded domains were unimportant, but that these skills were not amenable to end-of-shift or clinical assessment. For example, procedural skills may be better measured using a procedure-specific checklist during a directly observed encounter than on a global clinical assessment tool. Response rates for these polls ranged from 83–100%, with a mean response rate of 90%. The group achieved consensus regarding assessment format on all included domains (agreement 68–97%). Participants agreed that all domains should be assessed using a rating scale, with the exception of professionalism, for which a combined dichotomous/narrative format was selected. Response rates on these polls ranged from 44–99% (mean 83%) (Table 2).

The final NCAT-EM contains eight domains of assessment (see Figure). Six clinical performance domains are measured using a four-point rating scale based on the Association of American Medical Colleges core Entrustable Professional Activities (EPA) and the EM Milestones.^6^,^11^ Professionalism is measured dichotomously, with space for narrative comments when concerns are identified. A final norm-referenced global rating item requires assessors to rate the student relative to other students. The tool is designed for both paper-based and electronic formats. While it is intended to be a comprehensive assessment of the clinical performance of students in the emergency department, it is also intended to be only one element of a comprehensive evaluation of a student’s performance on an EM clerkship. Data from this tool should be used in concert with other assessment methods such as written exams, objective structured clinical examinations, direct observation sessions, presentations or projects, etc. when calculating a final grade.

Consensus guidelines for use of the NCAT-EM based on the results of Day 1 were these:

1. Faculty and senior EM residents should be the primary users of this tool
2. Assessors should undergo training prior to using the tool for assessment
3. The form should be used to assess performance on a single ED shift
4. No fewer than six independent assessments should be completed to translate the data into a grade
5. The tool may be used for all learner levels in the ED
6. Data from the form can be used to contribute to grades, SLOEs, and determination of competency.

## DISCUSSION

Based on the variability of current clinical assessment tools and practices, we anticipated large variability in opinion on the topics presented. However, we were surprised by the amount and strength of consensus on most topics, which likely reflects recognition among participants of the inadequacy of current assessment processes, and a desire to improve reliability, validity, and standardization across institutions. Overall, participants agreed on a large number of the themes and domains of assessment presented.

There were several pedagogical issues that arose during the consensus conference and post-conference Delphi process. While it is possible to assess medical knowledge with a clinical assessment tool, participants felt that it was much more important to assess the *application* of medical knowledge clinically. This was considered and voted on as a stand-alone domain of assessment, but the group agreed to exclude application of medical knowledge as its own domain as participants felt it was best incorporated into other domains (ability to generate a differential diagnosis, ability to generate a management plan, etc.). As a result, the application of medical knowledge is included in the anchors for multiple domains.

Qualitative input from participants strongly supported keeping the assessment tool as concise as possible to improve usability and response rate. To that end, post-conference work included discussion on whether to combine “Ability to Generate a Focused Differential Diagnosis” with “Ability to Generate a Management Plan,” and “Patient-centered Communication” with “Team-centered Communication.” The Delphi group did not reach a high level of consensus on either of these topics. Ultimately, the group decided to retain “Ability to Generate a Focused Differential Diagnosis” and “Ability to Generate a Management Plan” as unique domains of assessment, and to combine “Patient-centered Communication” with “Team-centered Communication,” while all other domains were retained as unique items on the assessment tool.

Another important conversation in the post-conference work was the reconciliation of the majority opinion during the conference to include elements of both peer-referenced and criterion-referenced assessment on the final form with the lack of consensus surrounding how best to frame a global assessment. This final element of the NCAT-EM was ultimately included as a peer-referenced element in order to provide at least one norm-referenced element of assessment.

Finally, professionalism is a domain of assessment for which a comprehensive, yet specific, assessment was problematic. “Professionalism” itself is a large, heterogeneous set of attributes ranging from punctuality to honesty to responsiveness to feedback. The NCAT-EM contains seven distinct professionalism attributes and an “other” category. This domain asks the assessor to identify whether there are concerns regarding any of the sub-domains, and if so, to describe them. This format implies that students by default exhibit professionalism unless otherwise noted. The group also felt that within each sub-domain, professionalism is an “all or nothing” proposition, and that any lapse merits serious consideration.

## LIMITATIONS

There were several limitations to this study. First, although widely publicized the conference was only attended by approximately one third of the CDEM Academy membership. Not all clerkship directors belong to the CDEM Academy or attend the CORD Academic Assembly, potentially biasing the results. However, it is likely that the educators who did participate are among the most engaged of the community with respect to clinical assessment of students in EM clerkships. Additionally, though they were not counted in the analysis and did not participate the day of the conference, many of the members of the theme groups were heavily involved in the preparation of the discussion. Residency leaders, non-physician educators, administrative professionals, students and other stakeholders were represented; however, it is unclear what the ideal ratio of participants may be. As this was a convenience sample, it may be that some minority groups were over- or under-represented.

Voting on the potential domains of assessment on Day 2 may have been affected by the order of presentation. Participants may have been more comfortable with the format and process after voting on the first few. Additionally, they may have been more apt to comment once they had a better understanding of the bigger picture and how the source materials were referenced. We attempted to mitigate this by providing the materials to participants beforehand and providing preparatory lectures to frame the questions and discussion at the beginning of both days of the conference. Finally, participants were able to change their vote as long as a poll was open. This affected the final results of some of the proposed domains as comments during the group discussion swayed votes. While this may be seen as a limitation in the study design, we feel this resulted in better representation of the group’s consensus.

## CONCLUSION

The NCAT-EM is the first national, standardized, consensus-derived, specialty-specific clinical assessment tool. The conference and the subsequent Delphi process leading to the development of this tool represent critical first steps in the development of national guidelines and a standardized approach to clinical assessment in undergraduate medical education. However, development of an assessment tool is only the first step. Critical next steps include measuring how the tool performs, comparison of the tool to existing assessments, development of training materials, and determination of how to implement its use.

Standardization of assessment practices across institutions will facilitate rigorous study of the reliability and validity of the tool itself, as well as enabling meaningful comparison of students across clerkships and institutions. The process of synthesizing the source data and seeking feedback from current clerkship directors could be emulated by other specialties, using their own specialty-specific source material, clinical priorities, and expert input. This may promote improved assessment of learners in other fields. While creation of a common assessment tool and guidelines were the primary objectives of our project, there was also an educational benefit for participants, who learned about current literature and best practices related to assessment, thus elevating the level of conversation around assessment in our specialty. Historically, clinical education research has been stymied by a lack of consistent assessment strategies and tools. Moving forward, the NCAT-EM has the potential to greatly improve educational research in EM, as well as improving the quality of learner assessment for the benefit of learners, educators, and ultimately patients.

## Figures and Tables

**Figure:**
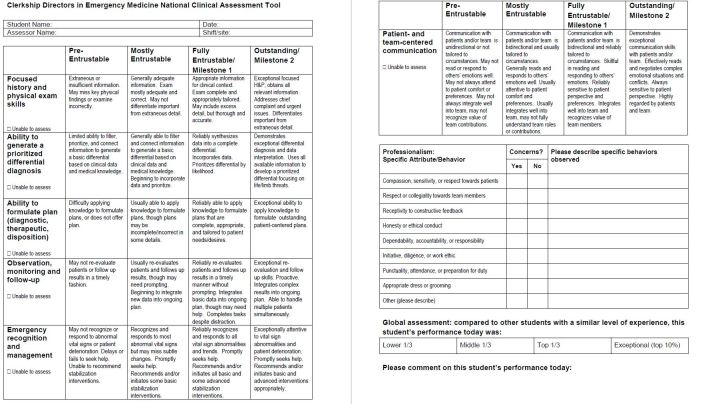
Clerkship Directors in Emergency Medicine National Clinical Assessment Tool.

**Table 1:** Leadership group for CDEM National Consensus Conference on Clinical Assessment of Medical Students in the ED.

					Current/past roles				
					
Rank	Name	Degrees	Institution	Rank	CD	UME director	APD	PD	Deanship
Executive committee	Douglas Franzen	MD, MEd	University of Washington	Assistant Professor	x	x	x		x
	Katherine Hiller	MD, MPH	University of Arizona	Professor	x	x			
	Julianna Jung	MD, MEd	Johns Hopkins University	Associate Professor	x	x			
	Luan Lawson	MD, MAEd	East Carolina University	Associate Professor	x	x			x
Theme leader, criterion- vs. norm-referencing	David Manthey	MD	Wake Forest School of Medicine	Professor	x	x			x
Theme leader, learners at different levels	Marianne Haughey	MD	CUNY Medical School	Professor	x		x	x	
	Joseph House	MD	University of Michigan	Associate Professor	x	x	x		
Theme leader, translation of assessment data	Matthew Tews	DO, MS	Medical College of Georgia	Professor	x	x			x
Theme leader, use of clinical assessment tools	Nicole Dubosh	MD	Harvard Medical School	Assistant Professor	x				
	Jonathan Fisher	MD, MPH	University of Arizona (Phoenix)	Professor	x	x	x		x
Theme leader, ensuring validation and research	David Wald	DO	Lewis Katz School of Medicine	Professor	x	x			x

CDEM, Clerkship Directors in Emergency Medicine; *ED*, emergency department; *CD*, clerkship director; *APD*, associate program director; *PD*, program director; *UME,* undergraduate medical education.

**Table 2:** Consensus regarding domains of assessment to include on a national end-of-shift assessment form in emergency medicine.

	Number voting	Agreement
Domains to include		
Ability to generate a prioritized differential diagnosis	64	98%
Format: rating scale	59	97%
Ability to formulate a management plan	61	97%
Format: rating scale	56	96%
Professionalism	58	97%
Format: combined dichotomous/narrative	65	88%
Global assessment	62	93%
Format: rating scale	59	86%
Format for rating scale: entrustability^*^	68	51%
Patient-centered communication^*^	58	83%
Format: rating scale	53	81%
Focused history and physical exam skills	66	77%
Format: rating scale	67	70%
Observation, monitoring and follow-up	64	75%
Format: rating scale	65	68%
Team-centered communication^*^	62	73%
Format: rating scale	31	87%
Emergency recognition and management	68	69%
Format: rating scale	69	70%
Domains not to include		
Resource utilization, ordering tests/consultation	67	90%
Problem-based learning and improvement	56	89%
Medical documentation	63	83%
Disposition	65	78%
Procedures	58	74%
No consensus		
Multitasking/task switching	71	62%
Medical knowledge	61	57%

## References

[1] Lawson L, Jung J, Franzen D (2016). Clinical assessment of medical students in emergency medicine clerkships: a survey of current practice. J Emerg Med.

[2] Khandelwal S, Way DP, Wald DA (2014). State of undergraduate education in emergency medicine: a national survey of clerkship directors. Acad Emerg Med.

[3] cordem.org [Internet] (c2016). Emergency Medicine Applying Guide.

[4] Love JN, Smith J, Weizberg M (2014). Council of Emergency Medicine Residency Directors’ standardized letter of recommendation: the program director’s perspective. Acad Emerg Med.

[5] Keim SM, Rein JA, Chisholm C (1999). A standardized letter of recommendation for residency application. Acad Emerg Med.

[6] aamc.org [Internet] Core entrustable professional activities for entering residency: Faculty and learners’ guide.

[7] Beeson MS, Carter WA, Christopher TA (2013). The development of the emergency medicine milestones. Acad Emerg Med.

[8] Manthey DE, Ander DS, Gordon DC (2010). Emergency medicine clerkship curriculum: an update and revision. Acad Emerg Med.

[9] Santen SA, Peterson WJ, Khandelwal S (2014). Medical student milestones in emergency medicine. Acad Emerg Med.

[10] Tews MC, Ditz Wyte CM, Coltman M (2015). Implementing a third-year emergency medicine medical student curriculum. J Emerg Med.

[11] acgme.org [Internet] The Emergency Medicine Milestones Project.

